# Vγ9Vδ2 T Cells: Can We Re-Purpose a Potent Anti-Infection Mechanism for Cancer Therapy?

**DOI:** 10.3390/cells9040829

**Published:** 2020-03-30

**Authors:** Klaus-Peter Künkele, Daniela Wesch, Hans-Heinrich Oberg, Martin Aichinger, Verena Supper, Christoph Baumann

**Affiliations:** 1 Boehringer Ingelheim RCV GmbH & Co KG, 1121 Vienna, Austria; klaus-peter.kuenkele@boehringer-ingelheim.com (K.-P.K.); Martin.Aichinger@boehringer-ingelheim.com (M.A.); Verena.Supper@boehringer-ingelheim.com (V.S.); 2 Institute of Immunology, University Hospital Schleswig-Holstein, Christian-Albrechts University of Kiel, 24105 Kiel, Germany; daniela.wesch@uksh.de (D.W.); Hans-Heinrich.Oberg@uksh.de (H.-H.O.)

**Keywords:** Vγ9Vδ2 T cells, gamma delta T cells, cancer, infection, BTN3A, phoshorylated antigens, drug development, immuno-oncology

## Abstract

Cancer therapies based on in vivo stimulation, or on adoptive T cell transfer of Vγ9Vδ2 T cells, have been tested in the past decades but have failed to provide consistent clinical efficacy. New, promising concepts such as γδ Chimeric Antigen Receptor (CAR) -T cells and γδ T-cell engagers are currently under preclinical evaluation. Since the impact of factors, such as the relatively low abundance of γδ T cells within tumor tissue is still under investigation, it remains to be shown whether these effector T cells can provide significant efficacy against solid tumors. Here, we highlight key learnings from the natural role of Vγ9Vδ2 T cells in the elimination of host cells bearing intracellular bacterial agents and we translate these into the setting of tumor therapy. We discuss the availability and relevance of preclinical models as well as currently available tools and knowledge from a drug development perspective. Finally, we compare advantages and disadvantages of existing therapeutic concepts and propose a role for Vγ9Vδ2 T cells in immune-oncology next to Cluster of Differentiation (CD) 3 activating therapies.

## 1. From Coley’s Toxin to Pattern Recognition Receptors

Famous first steps in immuno-oncology were undertaken by William Coley, who attempted to treat cancer patients by administration of *Streptococcus pyogenes and Serratia marcescens,* later described as Coley’s toxin [1]. Even though clinical statistics were not performed at today’s scale, Coley left an amazing amount of data to suggest that although this treatment resulted in severe toxicity, cancer could be treated and even cured by bacterial infection. Although subsequent therapeutic discoveries have led to the emergence of alternative approaches to treat cancer, including radiotherapy, chemotherapy and targeted therapies [2], bacterial treatment has been further evaluated in the clinic. Bacille Calmette-Guerin (BCG) is a Food and Drug Administration (FDA)-approved live attenuated preparation of *Mycobacterium bovis* (TICE®, Organon Teknika Corp. or PACIS®, BioChemPharma) and currently used as a standard immunotherapy for the treatment of bladder cancer [3]. 

Research of the past decades has drawn a map of a highly diversified and sensitive alarm system that has evolved to detect non-self structures of pathogenic intruders or molecular indicators of damaged cells. Pioneering work by Charles Janeway and Ruslan Medzhitov predicted and subsequently identified the first pathogen pattern recognition receptors and highlighted the importance of the innate immune system in the overall immune response [4]. The innate immune system recognizes pathogen-associated molecular patterns (PAMPs) or endogenous damage-associated molecular patterns (DAMPs), e.g., derived from essential bacterial functional or structural components, such as RNA or cell wall lipopolysaccharide (LPS) [5]. In the 21^st^ century, the responsible mediators and mechanisms of the anti-tumor activity of Coley´s toxin were identified: bacterial DNA and LPS stimulate Toll-Like Receptor (TLR) 9 and TLR4 signaling on a variety of immune cells, including Natural Killer (NK) cells and T cells, thereby enhancing their anti-tumor activity [6,7,8]. Additional studies have led to pathogen sensors being exploited therapeutically as drug targets to trigger a pro-inflammatory immune response [9,10]. Drug candidates acting as agonists of TLRs, of nucleotide-binding oligomerization domain (NOD)-like receptors (NLRs), or of stimulator of interferon genes (STING) entered the clinics with the aim to increase immune cell activation, infiltration and anti-tumor responses [11]. 

## 2. BTN3A1 is a PAMP Receptor

**Figure 1:**
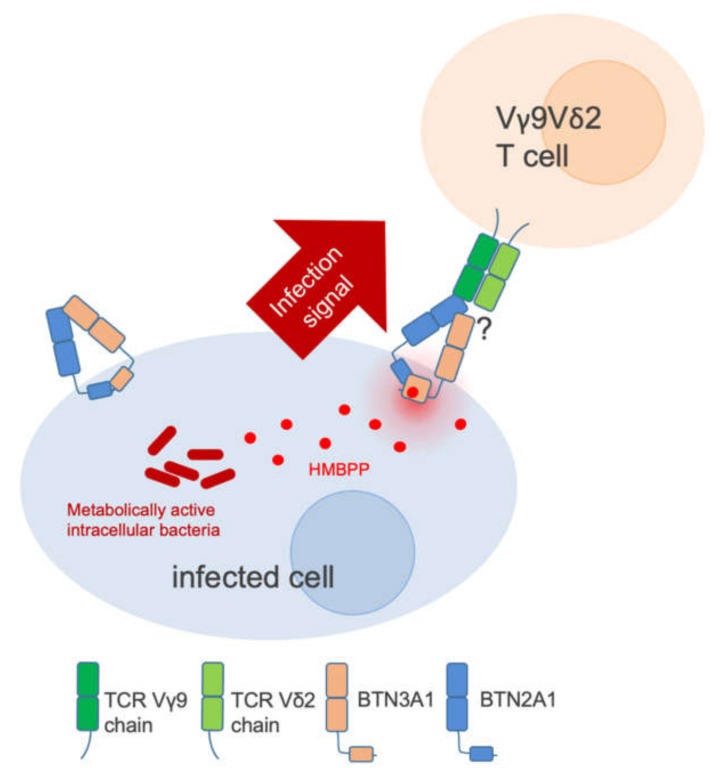
Butyrophilin 3 family member A1 (BTN3A1) is a pathogen-associated molecular pattern (PAMP) receptor. HMBPP from metabolically active bacteria, leaks into the cytoplasm where it is detected by the intracellular B30.2 domain of BTN3A1, triggering a conformational change within the protein. Thereby, the intracellular infection signal is transmitted through the plasma membrane to the surface, where BTN3A1 is constitutively associated with the Butyrophilin family member BTN2A1. Together they form an immunological synapse in which BTN2A1 interacts with the Vγ9 chain of the Vγ9Vδ2 TCR.

Butyrophilin 3 family member (BTN3) A1 is a transmembrane receptor that harbors two extracellular Immunoglobulin (Ig) -like domains and an intracellular B30.2 domain. The intracellular domain interacts directly with the bacterial metabolite (E)-4-hydroxy-3-methyl-but-2-enyl pyrophosphate (HMBPP) [12,13,14]. HMBPP is an essential intermediate product of the prokaryotic non-mevalonate/ 2-*C*-methyl-D-erythritol 4-phosphate/1-deoxy-D-xylulose 5-phosphate (MEP) pathway for isoprenoid synthesis, and is not generated by the mevalonate pathway, the human equivalent. BTN3A1 is exquisitely tuned to recognize this pathogen-derived molecule comparable to how TLRs recognize conserved pathogen structures, such as LPS or DNA [15,16,17]. The most intriguing difference, however, is the inside-out mode of action, where BTN3A1 senses its ligand in the cytosol and translates the signal to the cell surface [14] (Figure 1). Sophisticated structure based modeling and molecular data has led to the proposal that binding of HMBPP to the cytosolic B30.2 domain of BTN3A1 transmits this signal to the outside of the cell as a combination of a conformational change and receptor clustering [18,19,20]. Although an additional HMBPP binding site in the extracellular part of BTN3A1 has been proposed [21], the key role of intracellular HMBPP is further supported by the finding that extracellular HMBPP is degraded by the ecto-ATPase CD39 [22], preventing Vγ9Vδ2 T cell activation in an in vitro setting. 

The functional consequence of a BTN3A1 infection alarm is the activation of Vγ9Vδ2 T cells. The detailed interface, and interactions of infected cells and the T Cell Receptor (TCR) on Vγ9Vδ2 T cells, have not been solved and remain one of the currently hot topics in the field. Recently, the discovery that the related butyrophilin, BTN2A1, is an essential co-factor for the interaction of BTN3A1 with the Vγ9 TCR chain (Figure 1) has added further details to our current picture of the molecular synapse. BTN2A1 thus might represent the long-sought factor X on human chromosome 6 whose absence in the mouse prevented the activation of transfected, human BTN3A by phosphoantigens in the murine system [23,24,25].

Even though the variable diversity joining (VDJ) recombination that generates the gammadelta (γδ) TCR can create an even higher degree of diversity than that observed with the αβ TCR, Vγ9Vδ2 T cells respond to defined phosphorylated antigens which clearly does not require such a diversity [26]. Recent work has identified a conserved, germline-encoded region within the TCR variable region called hypervariable region 4 (HV4), which interacts with Butyrophilin family proteins and acts as a TCR intrinsic signal receiver. Therefore, it appears that the γδ TCR is able to recognize two different signals [27]: an innate (pathogen derived) signal and an adaptive signal, which has not been identified yet, but which requires TCR diversity.

## 3. Vγ9Vδ2 T Cells in Infection

While γδ T cells comprise 1%–10% of human Peripheral Blood Mononuclear Cells (PBMCs), Vγ9Vδ2 T cells are the major subset of γδ T cells in peripheral blood, and make about 60%–95% [28]. In support of the proposed mode of action of phosphoantigen response, Vγ9Vδ2 T cells have been associated with the host defense to infectious diseases caused by intracellular pathogens, such as malaria [29], visceral leishmaniosis [30], listeriosis [31], tularemia [32] and tuberculosis [33,34]. All these pathogens share a common feature of using the non-mevalonate pathway for isoprenoid synthesis. Stimulation of human PBMCs with *Mycobacterium tuberculosis (M. tb.)* preparations [35] and infection of monocytes by *M.tb.* or *Salmonella,* demonstrated that pathogen infection and phosphorylated antigens can activate and trigger expansion of Vγ9Vδ2 T cells [36]. Furthermore, strong Vγ9Vδ2 T cell responses against *M.tb.* and *Listeria monocytogenes* infected host cells were demonstrated in in vivo infection models in macaques [37,38]. 

## 4. Vγ9Vδ2T Cells in Cancer 

Bioinformatic analyses of large meta-genomic datasets determined the relative abundance of Vγ9Vδ2 T cells within tumors and correlated this with patient outcome. Tumor-infiltrating γδ T lymphocytes (γδ TILs) were found in all tumor entities, albeit at low numbers. Importantly, a correlation between relative abundance of γδ TILs and favorable response to immune checkpoint therapy in a variety of cancers was demonstrated [39,40]. 

Transformation can lead to differential expression or re-location of molecular signals to the surface of cancer cells and entail recognition by the immune system. Vγ9Vδ2 T cells have been shown to be able to recognize these markers of stress, including TCR ligands F1-ATPase in complex with apolipoprotein A-I, or hMSH2, a DNA mismatch repair protein [41,42]. 

It has been speculated that dysregulation of the mevalonate pathway during tumorigenesis is able to generate high intracellular levels of isopentenyl pyrophosphate (IPP) and that Vγ9Vδ2 T cells can sense this increase via signals to the BTN3A receptor [43]. IPP is the human metabolite most structurally homologous to bacterial HMBPP and has been shown to be able to trigger Vγ9Vδ2 T-cell activation and proliferation in an in vitro setting. It must be noted these effects required considerably higher concentrations (10000- to 30000-fold) of externally added IPP compared to the bacterial metabolite [44]. Although the intracellular IPP levels in cancer cells could be accumulated by up to 960-fold via downstream inhibition of farnesyl-diphosphate synthetase (FDPS) using aminobisphosphonates [45,46], without this intervention, only minimal cell killing was observed when Vγ9Vδ2 T cells were cultivated with a panel of tumor cells [47,48,49,50]. This indicates that natural IPP levels in most transformed cells may not be sufficient for activation of Vγ9Vδ2 T cells.

Importantly, in the presence of soluble BTN3A1 agonists, Vγ9Vδ2 T cells have been shown to be highly effective in cancer cell killing in co-culture experiments at various target: effector ratios. This requires BTN3A1 activation on tumor cells as pre-incubation of a BTN3A1 agonist with the effector T cells before addition to the target cells does not lead to tumor cell lysis, even at high T-effector: cancer cell ratios [13]. The data are further supported by another study showing that infected cells are much better activators of Vγ9Vδ2 T cell activity than heat-killed bacteria themselves [36]. This finding implies that an efficient target elimination signal must be traceable back to the infected/target cell. Indeed, Vγ9Vδ2 T cells build a synapse with the signal submitting target cell, which allows selective elimination of cells that harbor intracellular infection.

Recent advances in the sensitive detection of pathogens revealed that tumors harbor a diverse set of bacteria, viruses and fungi [51,52], a community, which, in analogy to the “microbiome” in the gut, has been labeled the “oncobiome” [53]. Sequencing of bacterial 16S RNA as a species-specific genetic fingerprint identified several hundred different bacterial species in each tumor [54]. There is increasing evidence that the oncobiome contains genes for the production and delivery of genotoxic molecules, of toxins altering the signaling pathways of the host cells, of enzymes metabolizing cancer therapeutics and of factors suppressing immune cell functions [55,56,57,58,59]. While the oncobiome, thus, has the means to drive tumorigenesis, progression, resistance and immune escape, the tumor cells on the other hand provide intracellular niches for bacterial growth. Tumor cells bearing activating mutations in the KRAS oncogene or phenotypically similar mutations show extensive macropinocytosis facilitating the transport of both, bacteria and nutrients, into the same intracellular compartments [54]. In contrast to extracellular bacteria that benefit from the death of tumor cells and the accompanying release of nutrients, e.g., in necrotic tumor regions, intracellular bacteria benefit from the survival of their proliferating tumor cell host. The host cell provides unlimited space and nutrition as well as protection against most immune cells—except Vγ9Vδ2 T cells, which are especially equipped to detect metabolically active bacteria occupying intracellular niches. It is, thus, tempting to speculate that HMBPP produced by bacteria of the oncobiome might be an important factor for the enrichment of Vγ9Vδ2 T cells in neoplastic regions and their positive impact on patient outcome.

## 5. Plasticity of Vγ9Vδ2 T Cells 

### 5.1. Cytotoxicity 

The strong cytolytic activity of Vγ9Vδ2 T cells is not only directed towards cells with intracellular bacterial infection but also against the pathogen itself. Effector functions of γδ T-cells includes induction of CD95/ CD95-Ligand and Tumor Necrosis Factor Related Apoptosis Inducing Ligand (TRAIL)/ TRAIL-Receptor (R) signaling to induce target cell apoptosis [60,61] and secretion of cytotoxic substances, including granzyme, granulysin and perforins, which are active against both the infected host cell as well as the pathogen [62,63,64]. Hence, the recognition of BTN3A activated by phosphoantigens can elicit cytotoxic effector functions of Vγ9Vδ2 T cells. In addition, stimulation through phosphoantigens and Interleukin (IL)-2 can upregulate the expression of FcγRIII (CD16) in circulating Vδ2 T cells [65], which when combined with therapeutic antibodies such as Rituximab or Obinutzumab can enhance the antibody dependent cellular cytotoxicity (ADCC) of Vγ9Vδ2 T cells and enhance their lytic activity on cancer cells, especially in hematological indications [66,67] and Neuroblastoma [68]. In another study, activated Vγ9Vδ2 T cells bound to Trastuzumab-treated breast cancer cells via CD16 and thereby exerted ADCC. The adoptive transfer of these activated Vγ9Vδ2 T cells together with Trastuzumab reduced growth of breast cancer tumors grafted into immune-compromised mice [69]. A very promising strategy to overcome certain limitations of Trastuzumab may be the use of the bispecific antibody [(HER2)_2_xCD16] which re-directs CD16^+^ γδ T cells to the tumor-associated cell surface antigen human epidermal growth factor receptor (HER)-2. The enhanced efficacy of [(HER2)_2_xCD16] can be explained by an increased degranulation and an enhanced cytotoxic activity against tumor cells which are resistant to CD95- or TRAIL-R induced cell death [70]. In sum, γδ T-cell cytotoxicity against tumor cells can be induced by different activation pathways and some of them may be more susceptible to immune suppressive mechanisms than others, depending on the specificity of the triggering non-self signal. Additionally, activation of Vγ9Vδ2 T cells induces a γδ T- antigen presenting cell (APC) phenotype and, thereby, the possibility to take up antigens including tumor antigens and to present them to αβ T cells [71,72].

### 5.2. Antigen Presentation

Recognition of tumor neo-antigens by T cells is fundamental to cancer immunotherapy and, hence, tremendous efforts are being undertaken to promote antigen presentation within the tumor [73]. Professional antigen presenting cells, in particular dendritic cells, are in the spotlight due to their ability to enhance local tumor recognition [74]. Strikingly, Vγ9Vδ2 T cells have been shown to be as potent as dendritic cells in their ability to present antigen to CD4^+^ and to cross present to CD8^+^ T cells [72]. Once they become activated, they either take up soluble antigen, as shown for the tumor antigen peptide Mart-1, or they phagocytose opsonized target cells [71,75]. After their activation, co-stimulatory molecules CD80 and CD86 as well as Major Histocompatibility Complex I (MHC-I) and MHC-II molecules are up-regulated on the surface together with the chemokine receptor CCR7, which directs the γδ T cells to the lymph nodes (LN); thus, increasing the likelihood that they interact with and activate CD4^+^ and CD8^+^ αβT cells [72]. 

In addition to their innate function, revealed by recognition and phagocytosis of pathogens and cross-presentation of antigens, γδ T cells rapidly produce cytokines and chemokines after activation, induce the maturation of dendritic cells, and have the capacity to kill various bacteria-infected Antigen Presenting Cells (APCs) and tumor cells in a HLA-non-restricted manner. Taken together, this illustrates their function as initiators of full-blown anti-infection adaptive immune responses as unique bridging cells between innate and adaptive immunity. 

### 5.3. Clonal Expansion

Even though Vγ9Vδ2 T cells constitute the largest population of γδ T cells in the peripheral blood, their abundance within CD3^+^ T cells in healthy donors is relatively low. In an infection setting, however, Vγ9Vδ2 T cells undergo massive expansion [76,77,78]. This has been best exemplified in Rhesus macaque models of *M. tb.* infection where initial pathogen challenge induced a strong Vγ9Vδ2 T cell-expansion over three weeks, followed by up to 1000x expansion of Vγ9Vδ2 memory T cells upon re-call infection with BCG [38]. In fact, in the presence of infection, Vγ9Vδ2 T cells can rapidly increase to represent up to 60% of peripheral T cells [79]. Most strikingly in this context is their ability to home back to the diseased and infected tissue after expansion. In *M. tb.* models, expanded Vγ9Vδ2 T cells exhibited trans-endothelial migration, interstitial localization and granuloma infiltration [80]. Similarly, Vγ9Vδ2 T cells increased drastically in numbers in non-human primate models of *Listeria monocytogenes* infection, expressed an effector memory phenotype and produced granzymes, perforin and pro-inflammatory cytokines [37]. Hence, the ability of these cells to rapidly expand to high numbers could be potentially exploited also in a cancer setting once appropriately stimulated.

### 5.4. Suppressor Functionality of γδ T Cells 

Similar to the functional diversification observed within CD4^+^ T cells, in vitro differentiation studies have brought forward the hypothesis that both tumor promoting and pro-inflammatory, anti-infective populations of γδ T cells exist [81]. On the one hand, activation of T cells by antigens in vitro results in secretion of cytokines, especially Tumor Necrosis Factor (TNF)-α and Interferon (IFN)-γ, and initiates pro-inflammatory, anti-infection responses [37]. On the other hand, Vδ2 T cells activated and expanded in vitro using CD28 agonistic antibodies, or by co-cultivation with IL-12 expressing dendritic cells, have been shown to exert suppressive functions on CD4^+^ T cells [82,83]. In fact, regulatory γδ T cells have been described that exhibit classical suppressive features, such as up-regulation of Programmed Death-Ligand (PD-L) 1 and secretion of Transforming Growth Factor (TGF)-β [84,85]. Hence, there seems sufficient evidence that inhibitory phenotypes of γδ T cells, including the Vγ9Vδ2 subpopulation, can be differentiated and described in vitro and ex vivo.

In primary tumors from patients with colorectal cancer, the bacterial induced activation of dendritic cells led to the polarization of γδ T cells into IL-17 producing γδ T cells, which recruited and induced the proliferation and survival of immunosuppressive polymorphonuclear myeloid-derived suppressor cells (PMN-MDSC) [86]. Of note, γδ T cells were detected with a pan TCR γδ antibody not permitting discrimination between γδ T cell sub-populations. 

Furthermore, the accumulation of Vδ1 Tregs in mammary tumors was correlated with poor survival [87] and in yet another study tumor infiltrating, tumor promoting γδ T17 cells have also been identified as Vδ1 T cells [86]. Interestingly, the clinical outcome of adoptive transfer of Vγ9Vδ2 T cells in patients with terminal breast cancer or hormone refractory prostate cancer correlated significantly and positively with sustained levels of Vγ9Vδ2 T cells in the peripheral blood [88,89]. Therefore, while it has been sufficiently elaborated that Vγ9Vδ2 T cells can potentially occur with suppressive features, their occurrence as such in primary tumors together with their implication for favorable outcome, might be different from other γδ T cell subpopulations and requires further investigation.

Despite the evidence of discrete populations of γδ T cells with opposite activities, a series of arguments speak in favor of harnessing the activity of Vγ9Vδ2 T cells to treat cancer. TGF-β secretion by Vγ9Vδ2 T cells is much lower than Vδ1 or CD4^+^ T cells and some reports suggest that TGF-β can actually enhance the cytotoxic activity of Vδ2 T cells [84]. Combination with TLR2, TLR3, TLR7 or TLR8 agonists abrogates the γδ T cells inhibitory effects in vitro and results in polarization towards a potent Th1 response [90,91,92,93]. Interestingly, activation of Vγ9Vδ2 T cells via phosphorylated antigens in monkey *M. tb.* in vivo models diminished IL-2 induced activation of CD4^+^ Tregs [94], suggesting BTN3A activation triggers predominantly an anti-suppressive effect. It is apparent that further studies are required to better understand the characteristics of γδ T cells in tumors and, especially, how their activation can be best exploited for cancer therapy [95,96]. 

## 6. Targeting Vγ9Vδ2 T Cells in Cancer Therapy

### 6.1. Adoptive T Cell-Transfer and In Vivo Stimulation

The capability of Vγ9Vδ2 T cells to undergo massive clonal expansion in infection settings is being exploited ex vivo using direct or indirect BTN3A agonists BrHPP or amino-bisphosphonates (such as zoledronic acid) in combination with IL-2. Many in vitro expansion protocols for γδ T cells have been established, opening the door for large-scale experiments, pre-clinical models and adoptive transfer studies [67,97] (Table 1). In the context of cancer therapy, it is now possible to highly enrich autologous cells by ex vivo expansion and re-infuse them into a cancer patient. This leads to elevated amounts of γδ T effector cells, which, otherwise, are not found in comparable abundance in cancer patients, especially when compared to the high numbers of CD8^+^ TCRαβ^+^ T-effector cells in the peripheral blood [64,98]. Subsequently, ex vivo expanded Vγ9Vδ2 T cells have been repeatedly used in early clinical Phase I adoptive transfer studies over the past 15 years (Table 1). The tolerability of adoptive transfer and low dose IL-2 was overall quite well and observed toxicities derived mainly from additional combination partners [99]. Even though the patient numbers are too low to draw significant conclusions on the efficacy side, it is interesting to note that in treatment schedules where Vγ9Vδ2 T cells were expanded ex vivo with the direct BTN3A ligand 2M3B1-PP, or where Zoledronate was co-administered in addition to Vγ9Vδ2 T cells, some partial or even complete remissions were observed [100,101,102]. Interestingly, expansion and survival of adoptively transferred Vγ9Vδ2 T cells in Colorectal Cancer (CRC) patients without co-infusion of supporting cytokines was working well and suggested promoting function of endogenous IL-2 or IL-15 [103]. Furthermore, in a pilot study with four patients who had different hematological malignancies, three complete remissions were achieved when half matched Vγ9Vδ2 T cells from family donors where transferred. Importantly, no graft versus host disease (GVHD) was observed [104].

Alternatively, clinical trials have also evaluated expansion of Vγ9Vδ2 T cells in the patient by administration of amino-bisphosphonates plus low dose IL-2. Reports from multiple clinical studies suggest that these approaches are well tolerated and, importantly, the clinical outcome was promising: in 3 out of 10 terminal breast cancer patients, two stable disease and one partial response have been observed [88] (Table 2). Despite the overall low patient numbers, in several studies a correlation between efficacy and the functionality and peripheral blood levels of Vγ9Vδ2 T cells was proposed [88,89,112]. Importantly, the peripheral blood numbers of Vγ9Vδ2 T cells could be increased, when IL-2 was co-administered to the patient [89]. Yet, repeated administration of IL-2 and Zoledronate seemingly reduced the effector cell levels suggestive of induction of anergy or activation induced cell death (AICD) [113]. Taken together, despite some signs of efficacy, the general patient response has been below expectations so far [114]. 

### 6.2. Chimeric Antigen Receptor T Cells (CAR-Ts)

The remarkable clinical response observed in patients with B-cell malignancies has led to FDA-approval of CD8^+^ CAR-T cells [118]. In CAR-T cells, a recombinant, chimeric αβ TCR molecule is introduced into the patient’s own CD8^+^ T cells to guide antigen specific tumor selectivity and to mimic an already expanded effector T-cell population.

One major limitation of this approach is the necessity to use the patient’s own T-cells, a personalized therapy that prevents the usage of allogeneic CAR-T cells as an off-the-shelf drug. This poses logistical hurdles to provide clinical-grade CD8^+^ CAR-T generation at a global level. Companies, such as Cellectis or Celyad, have addressed this issue by developing technologies to knock out the endogenous αβ TCR, thereby achieving allo-tolerability. Alternatively, CAR-T therapies are under development using γδ T cells, which circumvent this issue by recognizing innate antigens in a MHC-independent manner [119]. Gadeta is testing the safety of TEG001 in patients with relapsed/refractory acute myeloid leukemia (AML), myelodysplastic syndrome or multiple myeloma. TEG001 consists of autologous αβ T cells genetically transduced with a γδ TCR. The γδ TCR is added to allow recognition of malignant cells. The extensive investment in this area (e.g., in companies such as Puretech and Medinet) demonstrates the excitement to identify approaches to generate new recombinant CAR-T modalities which will reduce the requirement for autologous cells, add γδ T cell effector functions or bring alternative methods of tumor cell recognition. It is apparent, however, that therapeutic success is highly dependent on the tumor directed cytotoxic activity of T effector cells and it remains to be seen how strongly this impacts the clinical success of different γδ CAR-T approaches. 

### 6.3. BTN3A Agonistic Antibody

One of the most important tools generated in BTN3A research is the 20.1 agonistic antibody, which is now moving towards clinical development. This antibody originally helped to elucidate the BTN3A mode of action, effectively activating the receptor without the requirement for intracellular HMBPP or equivalent ligand binding [120,121]. Preclinical efficacy has been shown in γδ T cell adoptive transfer models of AML [122] and subsequently, Imcheck Therapeutics has introduced ImCheck Therapeutics (ICT)-01, a humanized BTN3A agonistic antibody, in Phase I trials in patients with hematological malignancies. 

### 6.4. T Cell Engager Approaches: γδ T Cell Engagers (γδ TcE)

Recognition of the tremendous potential of tumor antigen targeted activation of CD3^+^ T cells [123] has driven drug development in this area. Activity observed in pre-clinical studies [124] and potent efficacy in clinical trials, including long-term remissions, has led to the approval of blinatumomab ([CD3xCD19] BiTE) for the treatment of acute lymphoblastic leukemia (ALL) patients [125,126,127]. Clinical development of TcEs in solid tumors has lagged behind hematological malignancies with most data being around Amgen’s solitomab, an EpCAM^+^ CD3 BiTE. Clinical development was discontinued following evidence of serious toxicities [128]. Learnings from this approach have led to the development of AMG757, a DLL3-CD3 BiTE molecule in Phase I clinical testing, where utilization of a more tumor selective anchor protein, DLL3, raises hopes for reduced toxicity (https://clinicaltrials.gov/ct2/show/NCT03319940). The suppressive milieu in solid tumors may also limit the clinical efficacy of TcE approaches [98,129]. Accordingly, combination treatments are being tested pre-clinically and in the clinics to tip the balance of activation from the suppressors to tumor cell killers [130,131]. 

Even though there are some differences in the receptor architecture, the γδ TCR ensemble is structurally similar to the αβ TCR, carrying a CD3 subunit that can potentially be activated by an agonistic antibody [132]. While CD3 agonistic antibodies can readily activate and expand Vγ9Vδ2 T cells in the absence of IL-2, activation via CD3 in the presence of IL-2 induced growth arrest and apoptosis. Notably, the same cytokine is commonly used to boost clonal expansion of Vγ9Vδ2 T cells in the presence of phosphoantigens [133]. Akin to CD3 T-cell engagers, bispecific antibodies such as the Tribodies [(HER2)_2_xVγ9] or [(HER2)_2_xCD16] demonstrated that significant cytotoxicity can be achieved when target and effector γδ T cells are linked [70,134]. LAVA therapeutics has developed this approach further, generating a pre-clinical pipeline of different tumor anchor binding molecules linked to γδ TCR engaging antibodies. In contrast to γδ CAR-Ts and adoptive transfer studies, γδ T cell-engagers have to deal with the initially low effector cell numbers present in the system. On the other side, similar to the effector to target synapsis formation induced by CD3 T-cell engagers, they provide an alternative way of guiding γδ T effector cells to their target cells to induce cytotoxic activity.

## 7. Vγ9Vδ2 T-Cell Functionalities in Tumor Targeting

Even though not all of the above-mentioned therapeutic approaches are primarily based on Vγ9Vδ2 T cells, some common features linked to successful anti-tumor therapy are nevertheless shared. First of all, it is important to have sufficient numbers of effector cells to eliminate a solid tumor, especially considering the relatively low relative number of T-effector cell to tumor cells in late stage cancer. Obviously, adoptive transfer of autologous or even “off the shelf” Vγ9Vδ2 T cells will provide T-effector cell numbers, which will match or top the levels reached in infection settings. It is however still an open question if their cytolytic activity could be triggered by the endogenous level of IPP in tumor cells or by the low number of intracellular bacteria present in a fraction of the tumor cells [55]. As discussed above, if a given therapy was equally effective in stimulating cell expansion as intracellular pathogenic intruder detection by BTN3A, it might be able to exploit the tremendous expansion potential of Vγ9Vδ2 T cells. It will be helpful to clarify whether numbers of peripheral blood and/or tumor Vγ9Vδ2 T cells correlate with treatment success, what the minimal requirement for eliciting a response would be, and if the immune response has to be boosted by the addition of cytokines, such as IL-2 or other immune stimuli.

**Figure 2:**
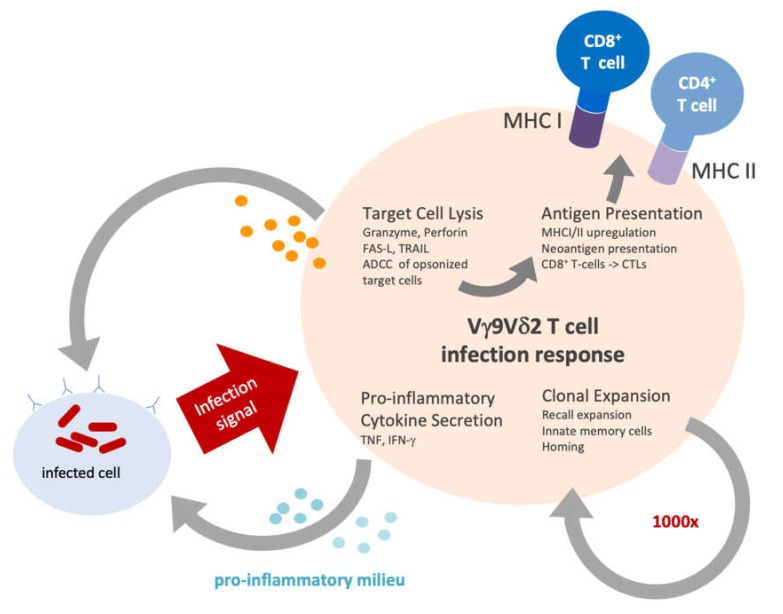
Immune functions of Vγ9Vδ2 T cells. An infection signal derived from an infected cell and mediated via BTN3A will induce a variety of cellular functions as follows:(i) lysis of the infected cell by cytotoxic mediators, such as granzymes, perforins or via the FS7-associated cell surface Antigen (Fas)/(CD95) and TRAIL-receptor induced cell death pathways; (ii) soluble material and opsonized, phagocytosed cellular material is digested and presented to TCR αβ^+^ CD4^+^ and CD8^+^ T cells; (iii) a clonal expansion up to 1000-fold increase after stimulation with their selective antigens; (iv) stimulated γδ T cells release many different cytokines and chemokines, most importantly the pro-inflammatory molecules TNF-α and IFN-γ.

It remains to be tested, which modality will be triggering which of the known Vγ9Vδ2 T-cell features (Figure 2). In addition to the direct cytotoxic activity, the concerted action of several effector functions, such as the ability to harness secretion of pro-inflammatory cytokines, antigen presentation or proliferation might contribute to the overall therapeutic outcome and long-term protection against the tumor.

## 8. Towards Precision Immune-Oncology

In contrast to hematological malignancies, solid tumors pose a greater problem in terms of immune biomarker determination. We still lack the detailed means to discriminate the immune cell subsets and their activation status in the tumor in a standardized fashion. However, recent efforts have come up with promising directions, such as the Immunogram [135], a collection of parameters describing the immune status, mutational burden, etc., of a tumor as an approach to identify tumors that would be responsive to immune-oncology therapies. Such an approach could be adapted to include T cell-subsets, such as Vγ9Vδ2 T cells, in the prediction of patient immune cell status and personalized therapy [136]. Indeed, Cibersort computational analyses have already shown that, with the right resolution, we might be able to discriminate and quantify immune cell subsets down to Vγ9Vδ2 T cells from sequencing data [39,40]. Other immuno-oncological (IO) biomarker approaches analyze tumor mutational burden or IFN-γ signatures and have been shown to have a high predictive value if used in combination [137]. In the end, the complexity of the immune system might require a comprehensive approach integrating multiple facets, including inflammation markers, epitope burden/neo-epitope occurrence, checkpoint receptor expression, suppressor cell populations, as well as T- cell-subset profiling to identify the right drug for the right patient [138]. 

Lessons from clinical trials might help to refine future patient selection biomarkers. Although Blinatumomab is considered the best therapeutic option for refractory ALL patients, it is important to note that 52% of treated patients show no response [139,140]. Biomarker analysis in a large sub-cohort of patients found the most significant biomarker correlating with patient response to be the number of CD4^+^ Tregs present in the peripheral blood (a cutoff of < 8.5% CD4^+^ Tregs in peripheral blood was able to identify 100% of responders and exclude 70% of non-responders) [141]. Subsequent ex vivo experiments with immune cell subsets purified from patient PBMCs demonstrated that lack of efficacy is caused by suppression of CD8^+^ T-cell activation. Indeed, the activation of CD3 on CD4^+^ Tregs by blinatumomab silenced the cytotoxicity of Cytotoxic T-Lymphocytes (CTL) [141]. Thus, as with other immune-oncology concepts, the success of CD3 activating antibody therapeutics relies heavily on the balance between suppressor cells and effector cells. Hence, in tumors where immune suppression does not allow for sufficient activation of effector T cells with a panCD3-activating compound, it might be more efficient to avoid further activating immune suppressive CD3 T cell subsets, but focus activation on smaller but specifically cytotoxic subsets. 

## 9. Preclinical Modeling

Mouse models have been invaluable in making complex physiological processes experimentally accessible [142]. Despite a lack of conservation of some aspects of immune biology between mice and humans, much of our current understanding of health and disease stems from, arguably, extrapolating results obtained in preclinical models to human biology. One of the more challenging aspects in this context is the exploration of γδ T cell function [143]. While the murine counterparts have been extensively studied in recent decades [144], strategies to study human cells in the mouse context remain experimentally more demanding. The reconstitution of immunocompromised murine hosts with selected cells of human origin, a process often referred to as “humanization”, is a widely used approach to study human cancer, human immune cells, but also many other human cell types under physiologic conditions [145]. 

Several studies, aimed at exploring the potential of Vγ9Vδ2 T cells as anti-cancer effectors (summarized in Table 3), have used similar strategies. The effect of adoptive transfer of in vitro expanded Vγ9Vδ2 T cells on the growth of established human tumors in immunocompromised mice (e.g., NSG, NOG, SCID, Foxn1^nu/nu^) was evaluated. In most studies, phosphoantigen-mediated activation and supplementation with growth-promoting cytokines have been employed to expand Vγ9Vδ2 T cells from PBMCs prior to adoptive transfer. Only few studies, however, have addressed the fate of adoptively transferred Vγ9Vδ2 T cells in mice. Although tracing studies suggest in vivo cell survival for periods of at least one to two weeks upon adoptive transfer [146,147], the functional activity of Vγ9Vδ2 T cells re-isolated from tumor bearing mice had vanished over time [147]. Hence, the requirement for high numbers and repetitive administration of human γδ T cells in these models may—at least in part—be ascribed to their limited persistence and functional activity in murine hosts [147]. Regardless, the sum of available data (Table 3) clearly demonstrate potent anti-tumor effector function of Vγ9Vδ2 T cells across indications and experimental setups [148]. 

Despite the variety of different cancer models and experimental approaches tested so far, it is apparent that repeated transfer of Vγ9Vδ2 T cells correlated with more pronounced tumor control. These conclusions are supported by an intracranial glioblastoma model [149], melanoma and pancreatic adenocarcinoma models [147], subcutaneous models of ovarian cancer [150], prostate cancer [151] and nasopharyngeal carcinoma [152].

The requirement for exogenous BTN3A ligands in Vγ9Vδ2 T cell mediated anti-tumor efficacy is less consistent. Although administration of bisphosphonates (e.g. zoledronate, alendronate or pamidronate) or 20.1 mAb [121] generally promoted Vγ9Vδ2 T cell mediated tumor killing, there appears to be some variability in the requirement for additional external stimuli. The majority of reports involving solid tumors suggest a stronger dependence on BTN3A agonism [50,147,149,150,151,153,154,155] than hematologic tumors [156,157,158,159]. 

From adoptive transfer studies in humans it has become apparent that Vγ9Vδ2 T cell mediated efficacy correlated with their proliferation, which was enhanced when IL-2 was administered [112]. Thus, while IL-2 is recognized as a crucial co-factor for Vγ9Vδ2 function and proliferation [160], the natural sources for IL-2 such as especially CD4^+^ Helper cells are absent in immunocompromised mice. Exogenous complementation of cytokine in murine models has, thus, been used in many experiments [134,147,161,162]. In contrast, studies that have not administered IL-2 in addition to the respective drug (Table 3) have also reported efficacy, suggesting that expanded and IL-2 pre-activated Vγ9Vδ2 T cells, which are repetitively administered, can be sufficiently active and viable in the mouse to mediate efficacy without cytokine addition.

**Table 3:** Summary of dual xenograft in vivo mouse studies involving Vγ9Vδ2 T cells. (i.v.) intravenous; (i.p.) intraperitoneal; (s.c.) subcutaneous.

Reference	Number of Transferred Cells	Route	Cell Source	Administration	*In Vivo* BTN3A Activation	Exogenous Cytokine Administration	Tumor	Mouse Strains
[122]	3 × 10^7^ Vγ9Vδ2	i.v.	PBMC	single	20.1 mAb	IL-15/IL-15ra (RLI)	Primary AML, U937	NSG
[161]	1 × 10^6^ γδ T cells	i.p.	γδTILs/TALs	single	none	IL-2	Daudi/SKOV3	BALB/c nude
[162]	2 x107 Vγ9Vδ2	i.p.	PBMC	repetitive	zoledronate	IL-2	MM1 CML	SCID
[163]	2 x10^7^ γδ T cells	i.v.	PBMC	repetitive	none	none	2LMP	SCID
[153]	5 x10^6^ Vγ9Vδ2	i.v.	PBMC	repetitive	zoledronate	no	SH-SY-5Y	BALB/c nude
[164]	5 × 10^6^ γδ T cells	s.c.	PBMC	single	none	no	NCI-H460	SCID
[154]	4 x10^7^ Vγ9Vδ2	i.v.	PBMC	single	alendronate	no	A375	SCID
[156]	4 x10^7^ Vγ9Vδ2	i.v.	PBMC	single	no	no	U937	NOG
[149]	1 x10^7^ Vγ9Vδ2	intracranial	PBMC	single and repetitive	zoledronate	none	U-87MG/orthotopic GBM	NSG
[147]	various	i.p.	PBMC	repetitive	alendronate	IL-2	MeWo PancTu1	SCID
[165]	2 × 10^6^ Vγ9Vδ2	i.v.	PBMC	repetitive	none	IL-2	Autolog. melanoma	CB.17 SCID
[146]	1 x107 Vγ9Vδ2	i.v.	PBMC	single	aledronate zoledronate	no	MDA-MB-231-hNIS.GFP	NSG
[157]	1x 10^7^ Vγ9Vδ2 enriched PBMCs	i.p.	PBMC	repetitive	none	no	Daudi	SCID
[150]	1 × 10^6^ Vγ9Vδ2	i.v.	PBMC	single and repetitive	pamidronate	no	OVCAR-3	NSG
[134]	1.5 -3 × 10^5^ Vγ9Vδ2	s.c.	PBMC	repetitive	Zoledronate [(Her2)_2_xVγ9]	IL-2	PancTu-I (PDAC)	SCIDbeige
[155]	1 x10^7^ Vγ9Vδ2	i.p.	PBMC	single and repetitive	aledronate zoledronate	no	SKOV-3 IGROV	SCID
[151]	1 × 10^6^ Vγ9Vδ2	i.v.	PBMC	single and repetitive	pamidronate	no	PC3	NSG
[158]	1 × 10^7^ PBMC +/- Vγ9Vδ2	i.v.	PBMC	repetitive	pamidronate	no	EBV induced B cell lymphoma	Rag2-/- γc -/-
[50]	1 x10^7^ Vγ9Vδ2	intravesicular	PBMC	single	zoledronate	none	UM-UC-3	SCID
[152]	5 × 10^7^ pan γδ T cells	i.v.	PBMC	single and repetitive	none	no	CNE2	BALB/c nude
[159]	1 x10^7^ Vγ9Vδ2	i.v.	PBMC	single	no	no	EBV induced B cell lymphoma	NSG

It is important to stress that only a very limited spectrum of Vγ9Vδ2 T cell biology can be faithfully addressed in these preclinical mouse models. While direct target cell killing can be readily assessed, the absence of accessory cells precludes examination of cellular functions described in Figure 2, such as antigen cross-presentation or production of pro-inflammatory cytokines.

An appealing way to bring complexity to tumor models while even maintaining immune cell diversity in a completely human setting are patient-derived ex vivo co-culturing experiments. Tumors of cancer patients removed during surgery were dissected and singularized tumor tissues as described elsewhere [64]. In brief, primary tumor tissue derived tumor cells and tumor-associated cells can be co-cultured with autologous isolated tumor-infiltrating cells (TIL) or autologous or allogeneic PBMC. Hence, other than in the above-described in vivo adoptive T-cell transfer in mouse studies, here the complexity of the tumor intrinsic immune system is largely maintained in an autologous, human system, which reflects the composition and phenotype of the different cells within the tumor tissue. Ex vivo cultivation of the original mixture of tumor cells and TILs with or without the addition of autologous PBMCs allows the more closely monitoring of the cytokine- or cytotoxic mediator expression using multicolor flow cytometry [64,134]. The cytotoxic activity of effector cells within the tumor tissue can be analyzed by applying the tumor tissue to a Real Time Cell Analyzer (RTCA, X-Celligence, ACEA Biosciences, San Diego, CA, USA), which allows measurement over an extended time course as described elsewhere [166]. Moreover, the assay enables the assessment of immune cell numbers and activation states by flow cytometry at the beginning and end of the experiment and its modulation upon pharmacological intervention. In contrast to the limited immune biology reflected in the adoptive transfer mouse models (see above)—the RTCA technology allows studying efficacy, immune cell phenotypes and their cross-talk at a timely resolution in an autologous, patient derived tumor setting. Hence, this methodology can be of great use when trying to reduce the translational gap between preclinical and clinical research and development.

## 10. Perspective

More than a century after the groundbreaking work of Coley, the connection between immune responses geared to fight bacterial infection and its potential for tumor treatment is more prominent than ever. An increasing number of infection sensors and immune regulators are being explored for cancer therapy. This is reflected in the continuously growing global immune-oncology drug pipeline, which is currently led by immune checkpoint targeting biologics like PD-1 antagonists [127,167].

Vγ9Vδ2 T cells are a fascinating member of the immune cell landscape because, in addition to sensing of antigens through the variable parts of their TCRs, they have developed the unique ability to detect a specific PAMP with the invariable parts of their TCRs. Through binding to activated BTN3A, they are able to detect the intracellular appearance of a metabolic intermediate of the bacterial MEP pathway. This is a particularly clever invention of nature: virtually all bacteria have this pathway and, therefore, it is a unique feature for clear discrimination between self and foreign. BTN3A triggering gives an unrestrained order for elimination of the infected cell, and diverse infection models, most prominently those using *Mycobacterium tuberculosis* or *Listeria moncytogenes* pathogens, have demonstrated the efficiency and versatility of γδ T effector cells. Recent breakthroughs, such as the elucidation of the BTN3A structure [13,18] or the 20.1 agonistic antibody [168], have provided mechanistic insight and tools and even prompted investigational therapeutic approaches. Not surprisingly, the pharmaceutical industry is increasingly interested, as reflected by the investments into the growing biotech field. However, as with all novel mechanisms, there is still a series of unknowns in the equation. In fact, the relative fraction of Vγ9Vδ2 T cells detected across tumor indications is relatively low [40] and it remains to be shown how they can be expanded to levels required for efficacy against a solid tumor. Moreover, it remains an open question whether system-wide activation will allow them to home in on and cross-link to tumor cells, which indeed is a pre-requisite for their elimination. Considering the pace at which the field is moving the answers might be at hand soon. 

From initial adoptive transfer clinical trials, we know that Vγ9Vδ2 T cells are tolerated but the trials so far have not resulted in a therapeutic response in a significant number of individuals [88]. Next generation approaches will build on Vγ9Vδ2 T cell biology and find improvements, either in optimizations of adoptive transfer, clever off-the-shelf CAR-T approaches, or biologics that cross-link the tumor to a γδ TCR. 

In order to reliably detect and combat infection, nature does not rely on just a single immune sensor trip wire. Widening our focus to other innate immune sensors might help to improve the outcome of sole Vγ9Vδ2 T cell-activation. TLR agonists have shown synergies and abrogation of suppressive states in vitro [92,93], and cytokines, such as IL-2, could be explored further to evaluate their synergistic effects on effector cell activation and expansion. On the other side, sensory pathways are being discovered which might give additional or alternative therapeutic angles, such as the recently identified MR-1 specific cancer-selective TCR clone [169]. Finally, while there are many hurdles to be jumped, γδ T cells with their high plasticity [27] represent a potential therapeutic option for a significant patient population with solid tumors. 

From an immunological perspective, cancer has multiple similarities with chronic infection, where an efficient anti-pathogen response is stalled by evasion mechanisms and T cell-exhaustion. It is therefore intriguing to consider novel therapies, not only by activation of a single immunological aspect, but rather by mimicking an acute infection scenario, thereby providing the means to install the complex, endogenous immune responses necessary to eliminate diseased cells. 

## Figures and Tables

**Table 1:** Completed Phase I clinical trials with adoptive transfer of ex vivo expanded, autologous Vγ9Vδ2 T cells. RCC: Renal Cell Carcinoma. MM: Multiple Myeloma. NSCLC: Non-small cell lung cancer. HCC: Hepatocellular Carcinoma. CRC: Colorectal Carcinoma. PR: Partial Response. SD: Stable Disease. CR: Complete Response. ^1^Bromohydrin Pyrophosphate (BrHPP). ^2^ Natural Killer (NK) cells. ^3^Cytokine Induced Killer Cells (CIK)

Reference	Indication	Treatment	*Ex Vivo* Expansion Stimulus	*n*	Response
[100]	RCC	γδ T cells	2M3B1-PP + IL-2 Teceleukin	7	3 PR
[105]	RCC	Innacell γδ T cells + IL-2	BrHPP^1^ + IL-2 Proleukin	10	6 SD
[106]	MM	γδ T cells	Zoledronate + IL-2	6	0
[107]	NSCLC	γδ T cells	Zoledronate + IL-2	10	3 SD
[101]	RCC	γδ T cells + Zoledronate + IL-2	2M3B1-PP	11	1 CR 5 SD
[108]	Diverse solid tumors	γδ T cells + Zoledronate	Zoledronate + IL-2	18	3 SD
[109]	Diverse solid tumors	γδ T cells + combinations	Zoledronate + IL-2	25	3 PR
[110]	NSCLC	γδ T cells	Zoledronate + IL-2	15	6 SD
[111]	HCC	Radiofreqency ablation + cytokines	NK^2^, CIK^3^, γδ T stimuli	30	
[103]	CRC	γδ T cells	Zoledronate + IL-2	6	
[102]	Gastric cancer	γδ T cells + Zoledronate	Zoledronate + IL-2	7	1 PR, 1 CR
[99]	Pancreatic Cancer	γδ T cells + Gemcitabine	Zoledronate + IL-2	28	
[104]	Hematological	γδ T cells (family donor) Zoledronate + IL-2	CD4^+^ and CD8^+^ T cell depleted PBMCs	4	3 CR

**Table 2:** Completed Phase I clinical trials with in vivo stimulation of gamma delta T cells. RCC: renal cell carcinoma, CRC: colorectal cancer. NHL: Non Hodgkins Lymphoma, MM: Multiple myeloma. OR: Overall Response. SD: stable disease. PR: partial response. AML: acute myeloid leukemia. Peripheral Blood Lymphocytes (PBL)

Reference	Indication	Treatment	*n*=	Response	Response Biomarker
[112]	Hematological (NHL + MM)	Pamidronate + IL-2	19	3 SDs	Vγ9Vδ2 PBL
[89]	Prostate Cancer	Zoledronate/Zoledronate + IL-2	18	1 SD, 1 PR 4 SD, 2 PR	TRAIL, Vγ9Vδ2 PBL
[115]	RCC, CRC, Breast Cancer	BrHPP + IL-2	28		
[88]	Breast Cancer	Zoledronate + IL-2	10	2 SD, 1 PR	Vγ9Vδ2 PBL
[113]	Metastatic RCC	Zoledronate + IL-2	12		Vγ9Vδ2 PBL
[116]	RCC, melanoma, AML	Zoledronate + IL-2	21	0 in solid tumors, 2 PR in AML	IFN-γ and in vivo expansion
[117]	Refractory neuroblastoma	Zoledronate + IL-2	4	1 SD	Vγ9Vδ2 PBL
